# Esophagus Dilation and Quality of Life in Adults with Scleroderma and Concomitant Obstructive Sleep Apnea

**DOI:** 10.3390/jcm13071884

**Published:** 2024-03-25

**Authors:** Tugce Yakut, Caner Cinar, Sait Karakurt, Haner Direskeneli, Yasemin Yalcinkaya, Yüksel Peker

**Affiliations:** 1 Department of Pulmonary Medicine, School of Medicine, Koc University, Istanbul 34010, Turkey; tugceyakut@gmail.com; 2 Department of Pulmonary Medicine, School of Medicine, Marmara University, Istanbul 34722, Turkey; dr.canercinar@gmail.com (C.C.); saitkarakurt@hotmail.com (S.K.); 3 Department of Rheumatology, School of Medicine, Marmara University, Istanbul 34722, Turkey; hanerdireskeneli@gmail.com; 4 Department of Rheumatology, School of Medicine, Istanbul University, Istanbul 34116, Turkey; yasemin_sahinkaya@hotmail.com; 5 Department of Molecular and Clinical Medicine, Sahlgrenska Academy, University of Gothenburg, 40530 Gothenburg, Sweden; 6 Department of Clinical Sciences, Respiratory Medicine and Allergology, Faculty of Medicine, Lund University, 22185 Lund, Sweden; 7 Division of Pulmonary, Allergy, and Critical Care Medicine, School of Medicine, University of Pittsburgh, Pittsburgh, PA 15213, USA

**Keywords:** systemic sclerosis, scleroderma, obstructive sleep apnea, quality of life, esophagus dilation

## Abstract

(1) **Background**: Systemic sclerosis (SSc) is a rare systemic disease, which often affects the esophagus, leading to dilation and complications such as dysphagia and reflux. Obstructive sleep apnea (OSA) is a chronic condition with recurrent episodes of upper airway collapsibility and is known to impair quality of life (QoL). The primary aim of this study was to investigate the occurrence of esophagus dilation in patients with SSc and concomitant OSA and, further, to address the impact of these conditions on QoL. (2) **Methods**: In this cross-sectional cohort study, 62 consecutive patients with SSc underwent chest computer tomography (CT) and home sleep apnea testing. The OSA diagnosis was based on AHI ≥ 15 events/h. The QoL was quantified using the short-form (SF)-36 questionnaire. The patients were dichotomized as high- vs. low-esophageal-diameter groups, based on the median cut-off values. (3) **Results**: The mean age was 48 ± 11 years; 58 (93.5%) were female; the mean BMI was 26.7 ± 5.0 kg/m^2^. The median esophageal diameter was 17.47 mm. A larger esophageal diameter was more frequently associated with the diffuse cutaneous subtype of SSc (*p* = 0.002) and significantly higher Warrick scores (*p* < 0.001), indicating more severe pulmonary fibrosis. There was a significant linear correlation between the Warrick score and the esophageal diameter (standardized β coefficient 0.544 [%95 confidence interval 0.250–0.609]; *p* < 0.001). In the subgroup analysis, the patients with both OSA and enlarged esophageal diameter experienced a significant decline in QoL, particularly in the domains of physical functioning, role physical, general health, role emotional, and vitality. (4) **Conclusions**: While OSA was not directly associated with enlarged esophageal diameter in patients with SSc, those with both OSA and enlarged esophageal diameter exhibited a significant decline in QoL. These findings suggest that the presence of OSA may exacerbate the adverse effects of esophageal dilation on QoL in SSc patients. Our results underline the importance of considering both gastrointestinal and sleep-related aspects in SSc management to enhance patient QoL.

## 1. Introduction

Systemic sclerosis (SSc) is a chronic autoimmune connective tissue disease affecting various organ systems, including the skin, lungs, heart, and gastrointestinal tract. The subsets of SSc include limited cutaneous SSc, diffuse cutaneous SSc, and SSc without skin involvement [1,2].

About 90 percent of patients with SSc have some degree of gastrointestinal (GI) involvement, and one of the most frequently affected regions within the GI tract is the esophagus. Approximately 50% of patients with GI involvement have symptoms such as dysphagia, reflux, and motility disorders [3]. These complications may significantly impair the quality of life (QoL) of affected individuals. Dysphagia and reflux cause physical discomfort and contribute to psychosocial challenges, including anxiety and depression symptoms, as well as further worsening patients’ QoL [4]. There is no effective treatment or cure for SSc, so the treatment for SSc is primarily aimed at managing symptoms, preventing complications, and improving QoL.

Despite the high incidence of esophageal complications in SSc patients, the concomitant occurrence of obstructive sleep apnea (OSA) is an area of concern that remains under-explored. Obstructive sleep apnea is a sleep disorder characterized by obstruction of the upper airway during sleep, leading to episodes of apneas or hypopneas [5]. This condition is associated with numerous symptoms, including excessive daytime sleepiness and cognitive challenges, which may further compound the GI symptoms, particularly reflux, experienced by these patients. Patients with OSA may complain of daytime sleepiness, morning headache, and memory and cognitive deficits [6]. Gastrointestinal symptoms, particularly GI reflux, are also common in patients with OSA [7]. Studies also indicate that untreated OSA significantly reduces QoL, affecting social, emotional, and physical well-being [8,9]. There are similar findings in other systemic rheumatologic diseases, indicating that reduced quality of life and sleep disturbances are common, further underscoring the effects of these conditions on patient well-being [10].

We previously demonstrated that SSc patients with concomitant OSA was associated with the risk of developing pulmonary hypertension, which was unrelated to the extent of pulmonary involvement. Scleroderma involvement of the lungs was shown in 40 out of 62 patients, and enlarged mean pulmonary artery diameter was present in 16 participants. Additionally, a significant correlation was identified between mean pulmonary artery diameter and OSA severity indices, namely the apnea–hypopnea index (AHI) and the oxygen desaturation index (ODI) [11]. It has been suggested that fibrosis associated with scleroderma may exacerbate OSA symptoms by affecting lung and airway function [12]. The systemic inflammation and fibrotic processes characteristic of SSc may predispose patients to both esophageal dilation and OSA. Given the close anatomical and physiological relationships between the esophagus and the upper airways, we also hypothesized that esophageal complications may interact with OSA in patients with SSc, potentially exacerbating both conditions and QoL. Thus, the primary aim of this study was to investigate the occurrence of esophagus dilation in SSc and its effect on QoL in patients with concomitant OSA in the same cohort.

## 2. Materials and Methods

### 2.1. Study Design and Participant Selection

This cross-sectional cohort study took place at the Marmara University Pendik Teaching and Education Hospital, Istanbul, during the period 1 April to 1 October 2016. All consecutive patients diagnosed with SSc and receiving care from the Departments of Rheumatology and/or Pulmonary Medicine departments, who did not have any known OSA, psychiatric or neurologic conditions, were invited to participate.

### 2.2. Basic Clinical Characteristics

Anthropometric information and past medical history for the whole cohort were extracted from patients’ medical files. Height and weight were measured, and body mass index (BMI) was determined using the ratio of body weight to the square of height in meters, with obesity identified by a BMI threshold of 30 kg/m^2^ or higher. Based on skin involvement scope, patients were sorted into categories of either limited cutaneous SSc or diffuse cutaneous SSc, following the criteria developed by Leroy et al. [13]. Baseline data on accompanying illnesses, such as hypertension and diabetes, were gathered through self-reporting or diagnoses confirmed by physicians. Current medications were also recorded.

### 2.3. Pulmonary Function Testing

Lung function tests involving forced vital capacity (FVC) and forced expiratory volume in 1 s (FEV1) was calculated with MIR Spirolab II spirometry (Medical International Research, Italy), diffusing capacity of lung for carbon monoxide (DLCO) was calculated using a body plethysmograph (CareFusion Type MasterScreen PFT; Hoechberg, Germany), and all results were analyzed following the established guidelines [14,15].

### 2.4. High-Resolution Computed Tomography

All patients underwent high-resolution computed tomography (HRCT) at the Department of Radiology, and Siemens SOMATOM Definition Flash (Erlangen, Germany) was employed for imaging. Scans older than six months were updated. The chest images were conducted with patients lying on their back and during end-inspiration. The area of the images covered from the top of the lungs to the bottom, with 0.6-millimeter collimation and slices ranging from 1 to 2 mm in thickness at increments of 0.5 mm. Five key anomalies were identified for assessment, and their seriousness was measured with the Warrick scoring system (Table 1) [16]. These included ground glass opacities (score 1), pleural irregularities (score 2), septal/subpleural lines (score 3), honey combing (score 4), and subpleural cysts (score 5). Additionally, an extent score was applied based on the number of lung segments involved: 1–3 (score 1), 4–9 (score 2), and more than 9 (score 3), respectively. The total possible score was 30.

The coronal esophageal diameter was assessed at 3 levels on CT chest scan, above aortic arch, between the inferior pulmonary vein and the aortic arch, and the diaphragmatic hiatus, respectively, by a senior pulmonologist blinded to the clinical data. The widest esophageal diameter was determined by measuring the greatest distance between inner esophageal mucosal limits at the 3 levels [17]. The patients were dichotomized into a high- or low-esophageal-diameter group, based on the median cut-off values.

### 2.5. Epworth Sleepiness Scale

The level of daytime sleepiness was assessed with the Turkish version of the Epworth Sleepiness Scale (ESS) questionnaire [18]. The ESS evaluates of 8 questions to assess the probability of falling asleep under 8 situations in the past month. Responses are rated on a scale from 0 to 3 (0, would never doze; 1 a slim chance; 2 a moderate chance; and 3 a high chance). The total ESS score can range from 0, indicating no sleepiness, to 24, the highest level of sleepiness. A score of 11 or higher on the ESS was categorized as excessive daytime sleepiness (EDS).

### 2.6. The Short Form 36 Health Survey (SF-36)

The Turkish version of the Short Form 36 Health Survey (SF-36) was used. It is a survey with 36 questions that represent eight health areas: physical functioning, role limitations due to physical health problems, bodily pain, general health, vitality, social functioning, role limitations due to emotional problems, and mental health. Each answer is transformed into a measure from 0 (worst) to 100 (best). Higher scores indicate better QoL. Additionally, patients’ SF-36 scores were compared with Turkish SF-36 normative data [19].

### 2.7. Home Sleep Apnea Testing

The simplified sleep study was performed through with a portable home sleep apnea testing (HSAT) device (NOX-T3; Nox Medical Inc., Reykjavik, Iceland), which included measurements. The device gauged nasal airflow with a pressure-sensitive cannula, detected chest and abdomen movement with plethysmography belts, and measured heart rate and blood oxygen saturation (SpO_2_) with a finger-pulse oximeter, as well as body position and movement. Additionally, the NOX-T3’s integrated microphone captured audio data for snore analysis. Patients with less than four hours of recorded sleep time were given the opportunity for new HSAT. An apnea was characterized as an approximately complete (≥90%) reduction in airflow, and hypopnea was noted as a drop in nasal pressure amplitude of ≥30% and/or thoracoabdominal movement ≥30% for ≥10 s if there was significant blood oxygen desaturation (a significant drop in SpO_2_ of at least 3% from baseline) according to the American Academy of Sleep Medicine’s latest guidelines [20]. Additionally, total number of significant desaturations was scored, and the oxygen desaturation index (ODI) was calculated based on the frequency of significant desaturations per hour of estimated sleep. Minimum SpO_2_ and time spent below 90% SpO_2_ (TS90%) were also noted. Obstructive sleep apnea was identified by an AHI of 15 or more events per hour, in accordance with the most recent sleep disorder classifications [21], without considering any accompanying OSA symptoms. All HSAT recordings were evaluated by a single physician (Y.P.), who was uninformed about patients, clinical details, and findings from the lung function tests and HRCT scans.

### 2.8. Statistical Analysis

For the analysis, variables were expressed as either the mean and standard deviation, or the median with the interquartile range, with 25th and 75th percentiles for continuous variables, and categorical variables were shown as counts and percentages. To compare baseline variables between groups, we used the independent-sample *t*-test or Mann–Whitney U for the continuous data and the chi-square test for the categorical data. Normality of the data was verified using the Shapiro–Wilk test. Kruskal–Wallis test was performed to compare the study subgroups, which were categorized relying on the combination of presence or absence enlarged esophageal diameter (EED) and OSA. Post hoc pairwise comparisons were conducted using the Mann–Whitney U test with Bonferroni correction.

A multivariate linear regression analysis was conducted to test variables associated with the primary outcome for the esophageal diameter. Covariates were chosen based on recent guidelines [22], including age, BMI, sex, disease duration, Warrick score, DLCO, time below SpO_2_ < 90%.

All statistical tests were two-sided, and a *p* value of less than 0.05 was considered significant. Statistical analysis was performed using SPSS version 26.0 for Windows (SPSS Inc., Chicago, IL, USA).

## 3. Results

**Figure 1:**
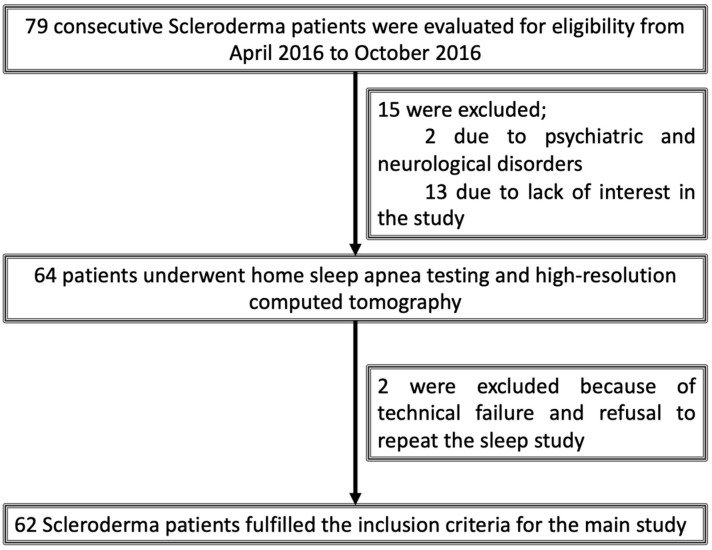
Flow chart of the study participants.

A total of 79 patients with SSc were identified as eligible (Figure 1). Two patients were excluded due to having accompanying psychiatric and neurological conditions, and 13 did not want to participate. Thus, 64 patients agreed and underwent HSAT. Two patients were excluded due to technical failure and 62 constituted the final analytic sample (43 with limited SSc, 19 with diffuse SSc). The mean age was 48 ± 11 years (range 21–72 years)), 58 (93.5%) were female, and the mean BMI was 26.7 ± 5.0 kg/m^2^. The median esophageal diameter was 17.47 mm, with a range of 7–39 mm in the entire cohort.

Table 2 shows the study cohort’s characteristics based on the diameter of the esophageal dilatation. We grouped the patients according to whether their esophageal diameter was < or ≥, with a median esophageal diameter of 17.47 mm. The patients with the greater esophageal diameters more often had dcSSc (51.6% vs. 12.9%, *p* = 0.002), and significantly higher Warrick scores (16.7 ± 9.5 vs. 7.65 ± 7.7, *p* < 0.001) than those with lower esophageal diameter. There were no significant differences between the groups regarding the pulmonary function test, HSAT findings, or medications (Table 2).

**Figure 2:**
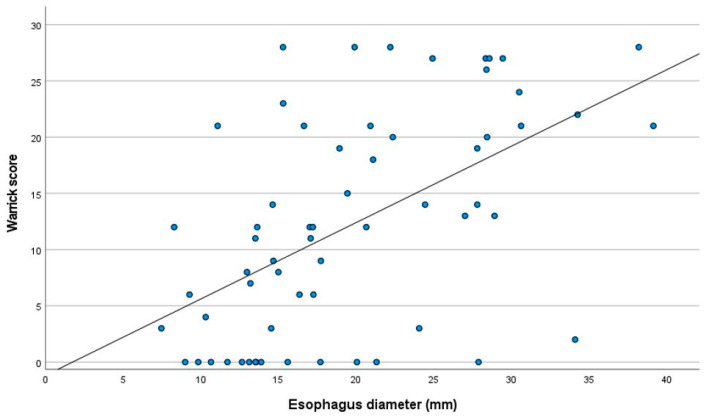
Correlation between the Warrick score and the esophageal diameter.

As shown in Figure 2, there was a significant linear correlation between the Warrick score and the esophageal diameter, which remained significant in the multivariate analysis adjusted for age, BMI, sex, disease duration, DLCO, and time spent below 90% in SpO_2_ (Table 3). None of the other tested variables showed a significant relationship.

**Figure 3:**
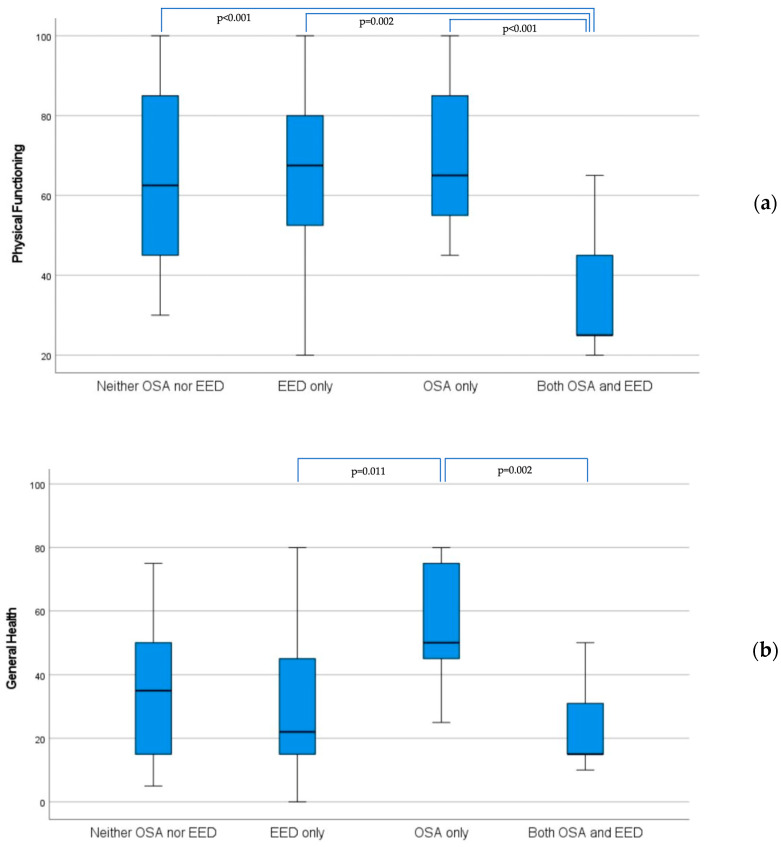
Comparison of the scores for physical functioning (**a**) and general health (**b**) on SF-36 Questionnaire for four subgroups of the study population.

Table 4 shows the respective SF-36 scores in the subgroups of patients with without OSA. There were markedly lower scores on five scales (physical functioning, role physical, general health, role emotional, and vitality) among the patients with enlarged esophageal dilatation with concomitant OSA, whereas those without OSA had no significant differences in their SF36 scores. As illustrated in Figure 3a,b, the patients with EED and concomitant OSA demonstrated the lowest physical functioning (a) scores when assessed with the other subgroups with EED or OSA alone, and with those with neither EED nor OSA. The patients with both EED and OSA demonstrated significantly lower general health (b) scores than those with only OSA. The cases with EED only also showed lower scores compared to the OSA-only group.

## 4. Discussion

The primary outcomes of our research were that OSA was not associated with an enlarged esophageal diameter in the patients with SSc, and that the patients with both OSA and enlarged esophageal diameter experienced a significant decline in QoL. This decline was particularly pronounced in the domains of physical functioning and general health, which are crucial aspects of daily living. These findings suggest that the presence of OSA may exacerbate the adverse effects of esophageal dilation on QoL in SSc patients. Thus, our results underline the importance of considering both gastrointestinal and sleep-related aspects in SSc management to enhance patient QoL.

To the best of our knowledge, this is the first study to report the effect of ED on QoL in patients with SSc and concomitant OSA. The correlation between enlarged esophageal diameter and decreased QoL in the presence of OSA could be indicative of more severe systemic involvement in SSc. The esophageal abnormalities may exacerbate the symptoms of OSA, leading to more pronounced sleep disturbances, which, in turn, can affect physical and mental health. Previous research showed a decrease in the QoL of SSc patients with gastrointestinal involvement. One of these studies demonstrated that GI symptoms negatively affected these patients’ QoL, particularly in social and emotional aspects, as measured by the Gastrointestinal Tract questionnaire [23]. Another study assessed the QoL of life in SSc patients and healthy controls using the SSC-GIT 1.0 instrument, focusing on social functioning and emotional well-being. A considerable number of SSc patients reported issues with social functioning and experienced lower levels of emotional well-being. Specifically, abnormal social functioning was found to be significantly common and severe in the SSc group compared to controls. No significant differences in emotional health were observed between the two groups or between SSc subtypes and disease duration [24].

Although it is known that OSA can reduce QoL, there is a lack of studies specifically investigating the impact of OSA on QoL in patients with SSc [9]. Nevertheless, previous studies investigated sleep disturbances within this patient group. The study shows that the prevalence of sleep disturbance among the patients was found to be 73.3%, and a significant association was identified between sleep disturbance and esophageal involvement. This association indicates a higher disease severity (as measured by the Medsger index). Patients with poor sleep quality experienced worse QoL, both mentally and physically, according to SF-12 results [25]. In another study, SSc patients reported worse scores on most MOS-Sleep scales, indicating poor sleep quality. Significant correlations were found between poor sleep quality and worsening pain, dyspnea, gastroesophageal reflux (as measured by the UCLA SCTC GIT 2.0), depression (CESD), and fatigue (FACIT-Fatigue) [26]. While these articles did not directly investigate the presence of OSA, the significant correlation between sleep disturbances and QoL factors is clear, suggesting that sleep quality is a critical component of patient care in SSc.

Numerous studies have established a relationship between esophageal dilation and interstitial lung disease in SSc. These studies provide convincing evidence of a positive correlation between increased esophageal diameter and the severity of ILD, as measured by radiographic scoring, along with a negative correlation with pulmonary function, forced vital capacity, and diffusion capacity for carbon monoxide [17,27]. Such findings support the hypothesis that esophageal abnormalities in SSc can lead to or worsen pulmonary complications, possibly via mechanisms such as micro-aspiration. This indicates that the role of esophageal involvement in SSc is complex and multifaceted, contributing to more than just dysphagia and reflux by playing role in the development of more severe lung disease. Currently, there is no definitive cure for SSc and, thus, the primary objective of treatment is to alleviate symptoms, prevent complications, and enhance QoL. For interstitial lung disease, the use of mycophenolate is recommended, and further research into the safety and efficacy of pirfenidone and the combination of pirfenidone with mycophenolate is encouraged. Additionally, the use of cyclophosphamide, rituximab, tocilizumab, nintedanib, and the combination of nintedanib with mycophenolate is suggested. For gastrointestinal symptoms, standard care includes lifestyle modifications to minimize the risk of occult micro-aspiration, with or without treatment with proton pump inhibitors, H2 blockers, and/or prokinetic agents [28]. Our study’s findings have significant implications for the treatment of SSc, particularly for patients presenting with both interstitial lung disease and gastroesophageal reflux. The observed correlation between esophageal dilation, OSA, and a decline in QoL underlines the need for a treatment approach that covers the full spectrum of SSc manifestations. Particularly in patients who present with gastrointestinal symptoms accompanied by sleep apnea symptoms, it is important to evaluate for OSA and, if necessary, incorporate appropriate treatment. Future studies should investigate the effects of these treatments on organ involvement to better understand and improve therapeutic approaches in SSc.

Lastly, our study’s emphasis on the absence of significant differences in SF-36 scores in the patients with or without enlarged esophageal diameter in the OSA-negative group could indicate that OSA might be a key modifier in the relationship between esophageal complications and QoL. It is important to consider the holistic management of these patients, focusing not just on the pulmonary implications, but also on the gastrointestinal and sleep-related symptoms to enhance their QoL.

## 5. Study Limitations

We should acknowledge several limitations in the current study. Firstly, the size of the study population was small, so the study may lack the statistical power needed to conduct subgroup analyses. Secondly, as the cohort was from a single center, the generalizability of the study’s findings may be limited. Although SSc is a rare disease, our study features one of the largest cohorts in the literature. Lastly, the HSAT, while a practical tool for diagnosing OSA in home environments, may not be as comprehensive as full polysomnography, and it may lead to the underestimation of the prevalence and severity of OSA. Nevertheless, a cutoff level of AHI 15 events/h was demonstrated to be reliable for OSA diagnosis with this kind of diagnostic tool such as HSAT when patients do not show typical symptoms of OSA [29].

## 6. Conclusions

While concomitant OSA is not directly correlated with ED in patients with SSc, it significantly compounds the decline in QoL for those with both conditions. This highlights the significant impact of esophageal dilation on QoL for patients with concomitant OSA, emphasizing an approach to patient management that includes both gastrointestinal and respiratory conditions. Further research is needed to confirm these findings and elucidate the underlying mechanisms between esophageal involvement, OSA, and QoL in SSc. Randomized controlled trials evaluating the effect of CPAP treatment on QoL in this group of patients are warranted.

## Figures and Tables

**Table 1:** Warrick scoring system.

Lesions and Lung Segments	Score
Pulmonary Abnormalities	Disease Severity Score
Ground-glass opacity	1
Irregularities at the pleural	2
Septal/subpleural lines	3
Honeycombing	4
Subpleural cysts	5
Number of Affected Segments	Disease Extent Score
1–3	1
4–9	2
>9	3

**Table 2:** Study population based on the diameter of esophageal dilation.

	Esophageal Diameter <17.47 mm	Esophageal Diameter ≥17.47 mm	
Variables	(*n* = 31)	(*n* = 31)	*p* Value
Age (years)	47.5 ± 12.7	49.4 ± 9.8	0.486
Female, *n* (%)	29 (46.8)	29 (46.8)	1.000
BMI (kg/m^2^)	26.4 ± 5.2	27 ± 5	0.788
Obesity, *n* (%)	10 (32.3)	11 (35.5)	1.000
Current smoker, *n* (%)	3 (9.7)	1 (3.2)	0.612
ESS score	5.8 ± 4.3	5.7 ± 5.3	0.492
Diffuse cutaneous SSc, *n* (%)	4 (12.9)	16 (51.6)	0.002
Hypertension, *n* (%)	6 (19.4)	5 (16.1)	0.740
Diabetes mellitus, *n* (%)	4 (12.9)	1 (3.2)	0.354
Cardiac disease, *n* (%)	2 (6.5)	2 (6.5)	1.000
HSAT findings			
AHI ≥ 15	9 (29)	11 (35.5)	0.587
AHI (events/h)	11.2 ± 10.2	14.5 ± 13.9	0.486
ODI (events/h)	9.2 ± 8.1	13 ± 12.8	0.410
Minimum SpO_2_ (%)	84.8 ± 6	83.5 ± 7.9	0.709
Time below SpO_2_ < 90% (min)	2.9 ± 5.7	6.9 ± 13.5	0.269
Mean SpO_2_ drops (%)	3.65 ± 0.8	3.8 ± 1.1	0.442
HRCT, spirometry, and DLCO findings			
Widest esophageal diameter	13.4 ± 2.7	25.9 ± 5.7	<0.001
Warrick Score	7.65 ± 7.7	16.7 ± 9.5	<0.001
DLCO (%) *	72.2 ± 20.9	66.7 ± 19.7	0.389
DLCO < 80% *n* (%) *	20 (74.1)	23 (73.3)	0.643
Medications			
Corticosteroids, *n* (%)	9 (29)	13 (41.9)	0.288
Methotrexate, *n* (%)	4 (12.9)	5 (16.1)	1.000
Hydroxychloroquine, *n* (%)	22 (71)	17 (54.8)	0.189
Mycophenolate mofetil, *n* (%)	4 (12.9)	11 (35.5)	0.073
Azathiopyrin, *n* (%)	4 (12.9)	10 (32.3)	0.127
Leflunomide, *n* (%)	2 (6.5)	0 (0)	0.492

Values are presented as mean ± standard deviation (evaluated with the independent student *t* test) or as the count of patients (percentage; analyzed using chi-squared test or Fisher’s exact test). Definition of abbreviations: BMI, body mass index; ESS, Epworth Sleepiness Scale; HSAT, home sleep apnea testing; AHI, apnea–hypopnea index; ODI, oxygen desaturation index; HRCT, high-resolution computed tomography; DLCO, diffusing capacity of lung for carbon monoxide. * Evaluated in 53 patients.

**Table 3:** Variables associated with esophageal diameter in adults with SSc.

	Standardized Coefficients Beta	95% Confidence Interval for Beta	*p* Value
Age	0.023	−0.138–0.170	0.955
BMI	0.118	−0.208–0.584	0.839
Sex	0.016	−5.913–6.871	0.881
Disease duration (years)	0.169	−0.058–0.459	0.126
Warrick score	0.544	0.250–0.609	<0.001
DLCO (%)	0.000	−0.101–0.102	0.988
Time below SpO_2_ < 90% (min)	0.126	−0.078–0.258	0.288

Abbreviations defined: BMI, body mass index; DLCO, diffusing capacity of lung for carbon monoxide; SpO_2_, oxyhemoglobin saturation.

**Table 4:** SF-36 scores in the subgroups of patients with SSc.

	Neither OSA nor EED (*n*:22)	EED Only (*n*:20)	*p*	OSA Only (*n*:9)	Both OSA and EED (*n*:11)	*p*
Physical Functioning	62.5 (45–86)	67.5 (51–80)	0.930	65 (53–85)	25 (25–45)	<0.001
Role Physical	62.5 (0–100)	37.5 (25–100)	0.886	100 (50–100)	25 (0–50)	0.006
Bodily Pain	63.5 (21–86)	52.5 (24–96)	0.649	65 (31–85)	45 (30–61)	0.230
General Health	35 (14–52)	22 (15–48)	0.587	50 (40–78)	15 (15–37)	0.001
Role Emotional	66.6 (33–67)	49.9 (8–67)	0.487	67 (50–100)	33.3 (0–67)	0.025
Vitality	35 (19–65)	42.5 (23–73)	0.528	60 (45–70)	35 (25–50)	0.007
Social Functioning	50 (38–100)	68.7 (48–100)	0.635	62.5 (44–94)	63 (25–75)	0.295
Mental Health	44.5 (37–68)	62 (37–84)	0.283	60 (48–69)	52 (36–72)	0.370

Definition of abbreviations: OSA, obstructive sleep apnea; EED, enlarged esophageal diameter. AHI ≥15 events/h was accepted as an indication of OSA. Esophageal diameter of ≥17.47 mm was accepted as an indication of EED.

## Data Availability

Individual participant data reported in this article can be provided by contacting the corresponding authors: tyakut@kuh.ku.edu.tr and yuksel.peker@lungall.gu.se.
